# An electrically conducting 3D coronene-based metal–organic framework†

**DOI:** 10.1039/d3ta07120k

**Published:** 2024-04-19

**Authors:** Marina I. Schönherr, Patricia I. Scheurle, Laura Frey, Marta Martínez-Abadía, Markus Döblinger, Andre Mähringer, Dominik Fehn, Lena Gerhards, Irina Santourian, Alfred Schirmacher, Tatjana Quast, Gunther Wittstock, Thomas Bein, Karsten Meyer, Aurelio Mateo-Alonso, Dana D. Medina

**Affiliations:** a Department of Chemistry, Ludwig-Maximilians-Universität (LMU) Butenandtstr. 11 (E) 81377 Munich Germany dana.medina@cup.lmu.de; b Center for NanoScience (CeNS) Schellingstr. 4 80799 Munich Germany; c POLYMAT, University of the Basque Country UPV/EHU Avenida de Tolosa 72 E-20018 Donostia-San Sebastián Spain; d Friedrich-Alexander-Universität Erlangen-Nürnberg (FAU), Department of Chemistry and Pharmacy, Inorganic Chemistry Egerlandstraße 1 91058 Erlangen Germany; e School of Mathematics and Science, Institute of Chemistry, Carl von Ossietzky University of Oldenburg 26111 Oldenburg Germany; f Physikalisch-Technische Bundesanstalt Braunschweig und Berlin (PTB) Bundesallee 100 38116 Braunschweig Germany; g Ikerbasque, Basque Foundation for Science 48009 Bilbao Spain

## Abstract

A novel cubic mesoporous metal–organic framework (MOF), consisting of hexahydroxy-*cata*-hexabenzocoronene (*c*-HBC) and Fe^III^ ions is presented. The highly crystalline and porous MOF features broad optical absorption over the whole visible and near infrared spectral regions. An electrical conductivity of 10^−4^ S cm^−1^ was measured on a pressed pellet.

## Abstract 2

A novel electrically conducting cubic mesoporous metal–organic framework (MOF), consisting of hexahydroxy-*cata*-hexabenzocoronene (*c*-HBC) and Fe^III^ ions is presented.

## Graphical abstract

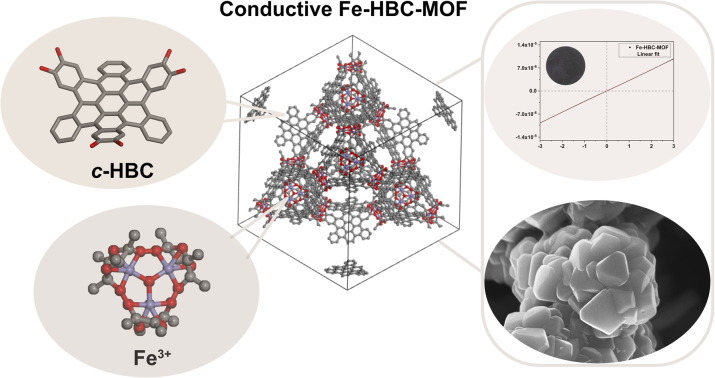

## 1. Introduction

Metal–organic frameworks (MOFs) are a prominent class of porous and ordered materials finding application in diverse fields such as gas storage, separations and catalysis.^1^ By adhering to the principles of reticular chemistry, the construction of MOFs with encoded properties has been the subject of significant research efforts over the past two decades, giving rise to a large number of reported new structures. In recent years, the intriguing property of electrical conductivity has been added as an exciting functional feature to the property portfolio of MOFs.^2^ The combination of long-range order, porosity, light absorption and charge carrier mobility may offer opportunities for applications in optoelectronics and (photo)electrocatalysis.^3^ The number of MOFs that possess this desired combination of properties is steadily growing, however the rational design of novel MOFs showing electrical conductivity is still a highly challenging task. For electrically conducting layered 2D MOF structures, charge migration can occur *via* both, through-space and through-bond mechanisms.^4^ In the context of electrically conducting 2D MOF structures, the use of hexahydroxytriphenylene (HHTP) as building block attracts great interest by virtue of its redox active nature and the planar aromatic backbone which favours stacking interactions. An early demonstration of layered HHTP MOFs is the metal-catecholate (M-CAT-1) series, establishing a prominent 2D MOF family which exhibit high electrical conductivity values ranging from about 0.1 S cm^−1^ to 1.5 S cm^−1^.^5^ Following the construction principle of the M-CAT-1 series, new electrically conducting 2D MOFs have been rapidly discovered and found to be suitable to serve in diverse fields of applications including, among others, chemiresistive sensing and electrocatalysis (Table S2†). In contrast, electrically conducting and porous 3D MOFs are still rather scarce.^6,7^ Notably, 3D frameworks can provide synthetic access to a large surface area – depending on the degree of interpenetration – and potentially offer enhanced molecular accessibility to reactive metal oxo sites. Recently, the rare-earth metal ions La^III^, Nd^III^, Ho^III^ and Yb^III^ have also been found to form electrically conducting 3D frameworks with HHTP.^8^ In that report, the HHTP ligand is held in tight molecular stacks being connected in-plane and out-of-plane by the respective metal ions. Hereby, different stacking distances corresponding to the specific lanthanide ionic radii are enabled giving rise to a variable electrical conductivity of up to 0.05 S cm^−1^. Prominently, the use of rare-earth metal ions Y^III^ and Eu^III^ as nodes in combination with HHTP afforded a cubic, porous structure, with electrical conductivity reaching 10^−5^ S cm^−1^ and surface area of up to 780 m^2^ g^−1^.^9^ Recently, novel cubic HHTP MOFs comprising Al^III^ and Ga^III^ metal ions, Al- and Ga-CAU-42, with high surface area have been reported.^10^ Along with that report, we demonstrated the synthesis of an HHTP MOF which is an iron analogue of the M-CAU-42 MOFs. Notably, the Fe-HHTP-MOF exhibits the desirable combination of high surface area of 1490 m^2^ g^−1^, long-range order, structural stability and electrical conductivity reaching 10^−3^ S cm^−1^ (Van der Pauw method, pellet).^11^ Moreover, the Fe-HHTP-MOF is a pitch black material featuring a broadband optical absorption ranging from 475 up to 1900 nm, and theoretical calculations indicated the structure to exhibit a continuous through bond charge-carrier pathway for electrons. Besides HHTP, other rigid molecules have been proposed as promising MOF construction elements. An intriguing molecular building motif is based on the rigid and conjugated coronene derivatives. To date, only a few coronene-based 2D MOFs have been reported, prominently a perthiolated coronene (PTC) containing Fe-PTC MOF that exhibits high electrical conductivity of 10 S cm^−1^ and a BET surface area of 210 m^2^ g^−1^.^12^ Additionally, a two-dimensional Ni-PTC MOF shows an electrical conductivity value in the same range.^13^ Non-planar aromatics have been recently introduced as novel structural building blocks for covalent organic frameworks (COFs).^14–16^ In particular, the rigid and contorted structure of *cata*-hexabenzocoronene derivatives gave rise to unique wavy 2D^14,16^ and trigonal trapezohedral 3D^15^ COF lattices. Recently, the use of catechol *c*-HBC derivative building blocks, possessing different connectivity vertices, has been extended to the construction of copper-connected 2D MOFs, where a certain MOF featured metallic-like charge transport.^17^ Employing a *c*-HBC derivative decorated with six catechol connecting groups afforded a 4-fold interpenetrated 3D MOF^18^ with an electrical conductivity of 10^−2^ S cm^−1^ (for el. conductivity values of iron- and coronene-based MOFs see Table S1†).

Herein, we report the synthesis of a novel 3D MOF, which is built with the rigid nonplanar building block 2,3,10,11,18,19-hexahydroxy-*cata*-hexabenzocoronene (*c*-HBC) connected by a trimeric Fe^III^ oxo-cluster, coined Fe-HBC-MOF. Similar to its prototype, Fe-HHTP-MOF,^11^ it crystallizes in a cubic symmetry as derived from X-ray diffraction analysis, structure modeling and electron microscopy. The Fe-HBC-MOF features a pore size at the boundary between microporous and mesoporous and a broad light absorption over the whole visible and near infrared spectral regions. The iron-valency was studied by means of ^57^Fe Mössbauer, X-ray photoelectron spectroscopy (XPS) and CW X-band EPR spectroscopy, indicating the sole presence of high-spin Fe^III^ in the structure. Van der Pauw electrical conductivity measurements revealed values of up to 10^−4^ S cm^−1^.

## 2. Results and discussion

### 2.1. Synthesis procedure

**Fig. 1:**
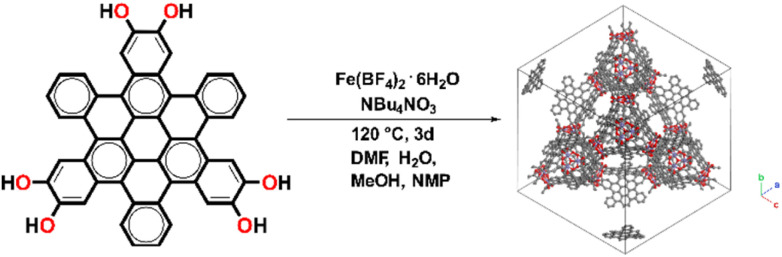
Schematic illustration of the solvothermal reaction of 2,3,10,11,18,19-hexahydroxy-*cata*-hexabenzocoronene (*c*-HBC) with Fe(BF_4_)_2_·6H_2_O as iron precursor, giving rise to the cubic Fe-HBC-MOF.

Fe-HBC-MOF was synthesized under solvothermal reaction conditions (Fig. 1). Briefly, the metal precursor Fe(BF_4_)_2_·6H_2_O, the ligand 2,3,10,11,18,19-hexahydroxy-*cata*-hexabenzocoronene (*c*-HBC) and the crystallization agent tetrabutylammonium nitrate (NBu_4_NO_3_) were dissolved in *N*,*N*-dimethylformamide (DMF), methanol (MeOH), *N*-methyl-2-pyrrolidone (NMP) and water under argon atmosphere (Fig. 1, details are given in the ESI†). The reaction mixture was sealed under inert conditions and treated by ultrasonication until complete dissolution of the reagents was achieved. Then, the sealed container was placed in a preheated oven at 120 °C for 72 h. After the designated reaction time, the container was allowed to cool down to room temperature. Subsequently, the product was washed with DMF, isolated under reduced pressure at room temperature and degassed under dynamic vacuum prior to analysis, yielding a deep-black microcrystalline powder. The activated MOF powder was found to be stable for more than a month under ambient conditions (Fig. S3†).

### 2.2. Structure description

**Fig. 2:**
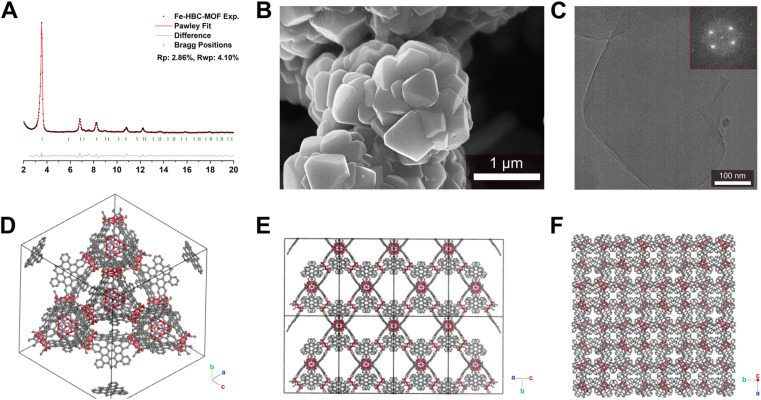
Experimental and Pawley-refined powder X-ray diffraction pattern of the Fe-HBC-MOF (A), SEM image of the octahedral intergrown crystallites with sizes up to 600–900 nm (B), TEM image of MOF crystallites. Inset: FFT taken for the crystallite examined (C) and display of the simulated structure of Fe-HBC-MOF with a view on the open supertetrahedron (D), view on the tetrahedral arrangement of the *c*-HBC ligands in the unit cell (E) and view along the *c*-axis showing the pore system (F).

Scanning electron microscopy (SEM) analysis of the powder obtained reveals faceted intergrown crystallites with a truncated octahedral habit (Fig. 2B and S13†), with edge-to-edge dimensions of up to 600–900 nm. Notably, the morphology of Fe-HBC-MOF is altered compared to the tetrahedral crystallites obtained for the Fe-HHTP-MOF prototype, however both are habits observed for cubic structures. The powder X-ray diffraction (PXRD) pattern of the reaction product displays sharp reflections up to high 2*Θ* values, confirming the formation of a highly crystalline material (Fig. 2A). Distinct reflections registered at 3.5, 6.8, 7.2, 7.6, 8.2, 9.0, 10.8, 12.3, and 13.7° 2*Θ* are observed in the PXRD pattern of a powder sample. The diffraction pattern of the Fe-HBC-MOF powder was indexed according to the cubic structure of Fe-HHTP-MOF (Fig. 2A and S6†). Using the unit cell of Fe-HHTP-MOF as a starting point, a model of the MOF unit cell was constructed (Fig. 2 and S7†). Thereby, the structure model exhibits construction elements similar to those of Fe-HHTP-MOF and matches the cubic space group *F*4_1_32. In the structure model, each iron metal is bridged by two bis-chelating *c*-HBC linkers. Three of these iron bis–catecholate complexes are held by a central oxygen atom, giving rise to a trimeric geometry (Fig. S8†). The iron oxo-trimers form the extended vertices of an open supertetrahedron, linked to three further iron oxo-trimers. The supertetrahedra are corner-connected to four other supertetrahedra, building up a porous superstructure (Fig. 2D). Subsequently, the model structure was refined using the Pawley method providing excellent refinement parameters (*R*_p_ = 2.86%, *R*_wp_ = 4.10%) and lattice parameters of *a* = *b* = *c* = 42.7 Å. In the diffraction pattern, the most prominent reflection at 3.5° 2*Θ* corresponds to the (111) reflection (Fig. S6†), in good accordance with a spacing of 25 Å obtained by transmission electron microscopy (TEM) and electron diffraction analysis (Fig. 2C and S5†). The TEM analysis of the Fe-HBC-MOF reveals large crystalline domains with a size of 350–450 nm. The Fast Fourier Transform (FFT) patterns obtained for the crystal lattice of two exemplary crystallites confirm a cubic structure (Fig. 2C inset), corresponding well with the PXRD pattern indexing and modeling data. Furthermore, compositional EDS analysis shows the presence of Fe, O and C (Fig. S5†). To further study the composition and the potential presence of guest molecules in the MOF structure, X-ray photoelectron spectroscopy (XPS) and elemental analysis (EA) were performed (Fig. S10–S12†). Through XPS, we confirm the presence of the elements Fe, O, C and N in the structure. The boron spectrum measured shows no signal, excluding the presence of impurities originating from the metal precursor, iron tetrafluoroborate. The nitrogen spectrum shows a weak signal, which is attributed to residual DMF or NMP in the sample. Moreover, an impurity of residual tetrabutylammonium nitrate can be excluded based on the XPS measurement (Fig. S11†). Furthermore, EA analysis of an activated Fe-HBC-MOF sample (see ESI†) indicated the presence of nitrogen, which cannot be correlated to the expected MOF chemical composition. Because Fe-HBC-MOF is structurally related to M-CAU-42 (M = Al, Ga), exhibiting a chemical formula of ((CH_3_)_2_NH_2_)_2_ [Ga_3_O(HHTP^6−^)(HHTP˙^3−^)]^21^ which accounts for the presence of nitrogen by dimethylammonium (DMA^+^) being part of the structure as countercation, we attribute the residual nitrogen to the DMF solvent utilized for the synthesis, in line with XPS results (Section 3 in the ESI†). For charge balancing, we propose that *c*-HBC exists in two oxidation states resulting in a chemical formula of ((CH_3_)_2_NH_2_)_2_ [Fe_3_O(HBC^6−^) (HBC˙^3−^)], similar to the chemical formula of Ga-CAU-42. Thermogravimetric analysis (TGA) manifests a multistep mass loss (Fig. S9†). Primarily, a prominent mass loss was initiated at 110 °C, associated with the loss of solvent molecules and structural degradation, followed by a second mass loss leading to a final decomposition of the MOF framework. The Fourier-transform infrared (FTIR) spectra complementarily indicate the framework formation, supported by the strong attenuation of the hydroxyl absorption band *ν*(OH) at 3400 cm^−1^ of the *c*-HBC linker molecule upon coordination to the iron ion (Fig. S14†).

**Fig. 3:**
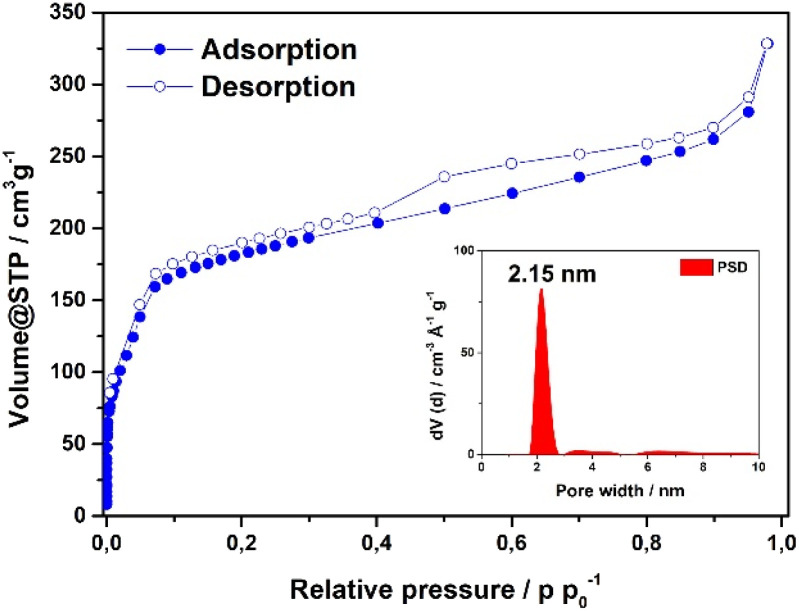
Nitrogen sorption isotherm of the Fe-HBC-MOF. The pore size distribution was obtained by quenched solid density functional theory (QSDFT) (shown as inset).

The accessible surface area and porosity of the Fe-HBC-MOF was determined by nitrogen physisorption at 77.3 K (Fig. 3). The recorded isotherm features the IUPAC type IV(b) characteristic for mesoporous materials,^19^ with a sharp first nitrogen uptake at relatively low partial pressures (*p*/*p*_0_ < 0.1 and up to 75 cm^3^ g^−1^) and a successive uptake of up to 160 cm^3^ g^−1^ attributed to capillary condensation in the mesopores (Fig. 3). Evidently, the desorption curve shows a small hysteresis and a reversible gas sorption process. The BET surface area was determined to be as high as 463 m^2^ g^−1^. Quenched solid density functional theory (QSDFT) calculations reveal a pore size of 2.15 nm (see inset in Fig. 3).

### 2.3. Photophysical properties

**Fig. 4:**
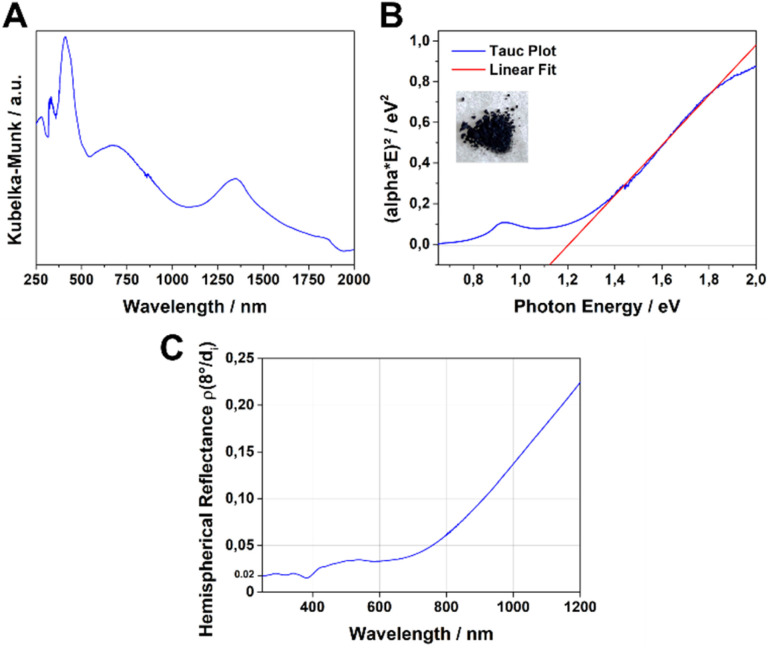
UV-Vis-NIR spectrum of the Fe-HBC-MOF (A), Tauc plot, assuming a direct band gap, showing a band gap of 1.20 eV for the Fe-HBC-MOF (B) and spectral reflectance measurement reveals absorption of 94% over the whole visible spectrum up to 800 nm (C).

The UV-Vis-NIR absorption spectrum of the Fe-HBC-MOF was measured for a powder sample in a diffuse reflectance geometry and evaluated using the Kubelka–Munk equation (Fig. 4A). Fe-HBC-MOF features a broad absorption over the whole visible spectrum into the near IR region, with maxima at 410 nm, 680 nm and 1340 nm. These absorption characteristics correspond to the apparent deep black colour of the MOF powder. Utilizing the apparent absorption onset at 1090 nm (and recognizing additional absorption features at lower energies), and by assuming a direct band gap, a band gap of 1.20 eV was calculated by constructing a Tauc plot (Fig. 4B). Interestingly, with steady-state photoluminescence (PL) measurements of the MOF (with an excitation wavelength of 378 nm) photon emission was not detected. In view of the broad-range absorption capacity of Fe-HBC-MOF, we conducted total hemispherical reflectance measurements to determine quantitatively the reflectance capabilities of the MOF. Fe-HBC-MOF shows a broad absorption covering almost the entire UV-vis and near-IR regions, yielding a total absorption of 94% up to 800 nm in the spectral region of visible light (Fig. 4C) and in line with the deep black appearance of the material.

### 2.4. Iron valency

**Fig. 5:**
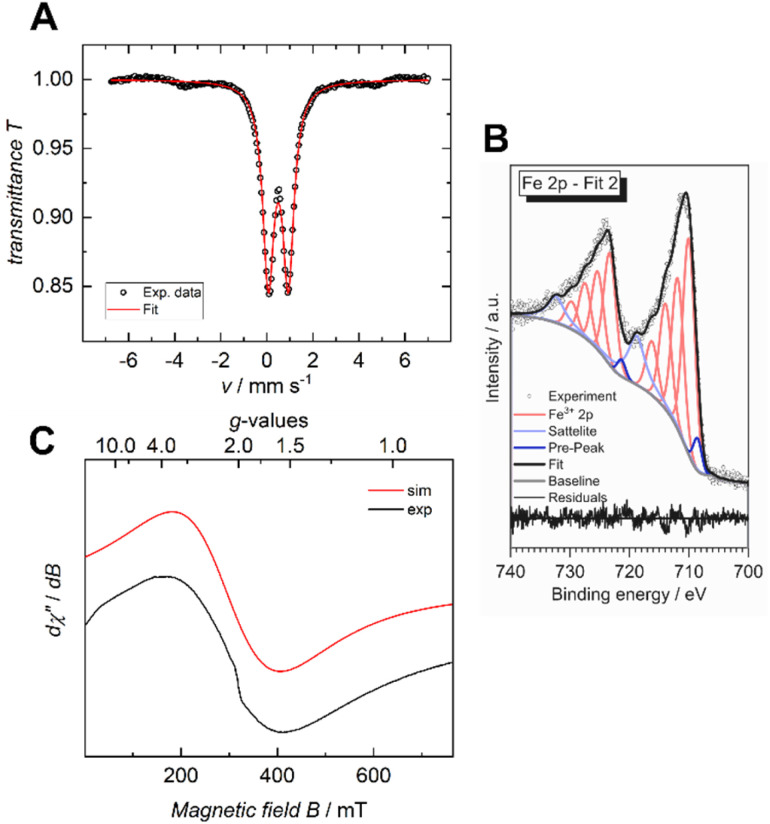
Zero-field ^57^Fe Mössbauer spectrum of Fe-HBC-MOF recorded at 77 K in the solid state (A), XPS spectrum of the Fe 2p region (B) and CW X-band EPR spectrum recorded at 293 K (C).

The iron valency is of importance for shedding light on the structural and electrical properties of the MOF, particularly in light of the fact that mixed-valance iron is reported to promote electrical conductivity in coordination polymers and MOFs.^7,20^ In the case of the previously reported Fe-HHTP-MOF, a ferric framework had been revealed. To determine the iron valency for the Fe-HBC-MOF, zero-field ^57^Fe Mössbauer spectroscopy was employed at 77 K. The spectrum shows one iron species with an isomer shift of *δ* = 0.50 mm s^−1^ and a symmetric quadrupole splitting, Δ*E*_Q_ of 0.87 mm s^−1^, characteristic for a single high-spin ferric Fe^(III)^ coordination site present in the MOF, and it confirms the absence of Fe^(II)^ precursor in the samples investigated (Fig. 5A and S16†). Also, XPS data recorded for the Fe-HBC-MOF agree well with the findings of the ^57^Fe Mössbauer spectroscopy. The recorded Fe 2p signal of the XPS measurement shows a peak splitting into two broad Fe 2p_3/2_ and Fe 2p_1/2_ peaks (Fig. 5B). The fitting of each peak by four components agrees well with the interpretation of the signals based on high-spin Fe^III^. This is in line with the iron valency analysed in the Fe-HHTP-MOF prototype. In addition, continuous wave (CW) X-band EPR spectra of the Fe-HBC-MOF powder were recorded at 95, and 293 K (Fig. S18†). At 293 K, the spectrum shows a single, broad (*W*_iso_ = 255 × 10^−4^ cm^−1^/GHz) and isotropic signal, centered at *g*_iso_ = 2.02 (Fig. 5C and S17†), which confirms the presence of Fe^III^ nuclei in the framework.

### 2.5. Electrical conductivity

**Fig. 6:**
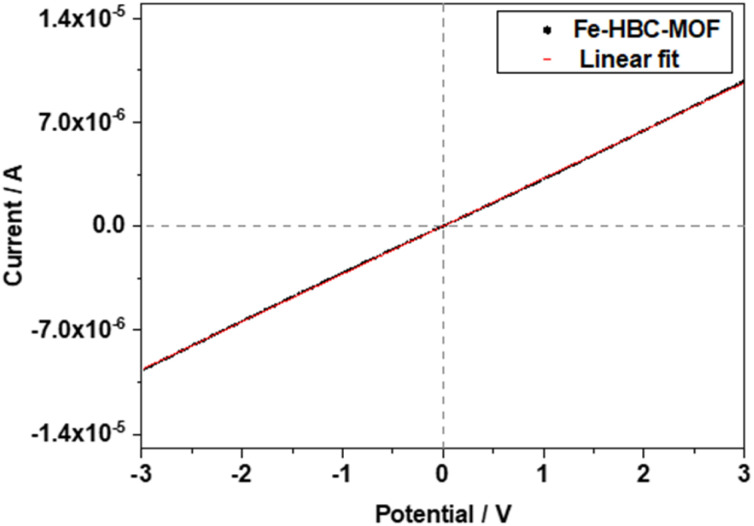
*I*–*V* curve of the Fe-HBC-MOF obtained *via* a two-probe measurement.

The electrical conductivity of the Fe-HBC-MOF was determined on crystalline samples using two-probe and van der Pauw measurements. For two-probe measurements, crystalline pelletized samples were employed (for further information see ESI,†Fig. 6). Under inert conditions, the pellets were placed between two brass electrodes of an in house-built instrument. The thickness of the pellets was determined with a slide gauge to be 130 μm (for further information see ESI†). Sweeping the voltage in the range of −3 to 3 V provided a linear ohmic resistance curve, which was fitted by a linear regression, giving an electrical conductivity of up to 3 × 10^−6^ S cm^−1^. The electrical conductivity of the same Fe-HBC-MOF pellet was also measured with the van der Pauw method, affording an average electrical conductivity of 5.5 × 10^−4^ S cm^−1^. These measurements were conducted under ambient conditions. Notably, applying pressure on crystalline MOF powders results in an evident decrease in crystallinity (Fig. S4†). To categorize the origin of the electrical conductivity observed in this MOF, we point to two main factors discussed in the manuscript: employing iron ions (with mixed valency capability) as coordination centres and conjugated, potentially redox active *c*-HBC ligands that act synergistically to enhance electrical conductivity in line with literature reports.^7,21^

## 3. Conclusions

Here, we established the successful synthesis of a novel cubic ferric coronene framework, named Fe-HBC-MOF. It was obtained as black powder in a solvothermal synthesis. SEM images reveal a morphology of faceted intergrown crystallites with a truncated octahedral habit and a size range of 600–900 nm. The simulated PXRD pattern of the structural model in a cubic space group matches well with the powder X-ray diffraction pattern recorded. According to the structural model, iron-connected supertetrahedra form a diamond-like topology. The ferric character was confirmed by means of XPS, CW X-band EPR and ^57^Fe Mössbauer measurements, demonstrating the presence of high-spin Fe^III^ in the framework. The Fe-HBC-MOF is a mesoporous material and shows electrical conductivity values of up to 10^−4^ S cm^−1^. In accordance with its deep-black colour, it features a broad absorption over the whole visible spectrum with up to 94% absorption (as powder) at this spectral range and in the NIR region, with a calculated band gap of 1.20 eV (assuming a direct transition) making the 3D Fe-catecholate MOF series suitable for potential applications in light harvesting and energy conversion.

## Conflicts of interest

The authors declare no conflict of interest.

## Supplementary Material

TA-012-D3TA07120K-s001
