# Supplementary material for: An electrically conducting 3D coronene-based metal–organic framework
Source: J Mater Chem A Mater. 2024 Apr 19;12(17):10044–9. doi: 10.1039/d3ta07120k (PMC11060507; doi:10.1039/d3ta07120k)
Supplement: TA-012-D3TA07120K-s001 [file TA-012-D3TA07120K-s001.pdf]

Electronic Supplementary Material (ESI) for Journal of Materials Chemistry A.
This journal is © The Royal Society of Chemistry 2024

Supplementary Information

An electrically conducting 3D coronene-based metal–organic framework

Marina I. Schönherr,[†a,b] Patricia I. Scheurle,[†a,b] Laura Frey,[a,b] Marta Martínez-Abadía,[c] Markus Döblinger,[a,b] Andre Mähringer,[a,b] Dominik Fehn,[d] Lena Gerhards,[e] Irina Santourian,[f] Alfred Schirmacher,[f] Tatjana Quast,[f] Gunther Wittstock,[e] Thomas Bein,[a,b] Karsten Meyer,[d] Aurelio Mateo-Alonso,[c,g] Dana D. Medina,[*a,b]

[a] Department of Chemistry, Ludwig-Maximilians-Universität (LMU), Butenandtstr.11 (E), 81377 Munich, Germany.

[b] Center for NanoScience (CeNS), Schellingstr. 4, 80799 Munich, Germany.

[c] POLYMAT, University of the Basque Country UPV/EHU, Avenida de Tolosa 72, E-20018 Donostia-San Sebastián, Spain.

[d] Friedrich-Alexander-Universität Erlangen-Nürnberg (FAU), Department of Chemistry and Pharmacy, Inorganic Chemistry, Egerlandstraße 1, 91058 Erlangen, Germany.

[e] School of Mathematics and Science, Institute of Chemistry, Carl von Ossietzky University of Oldenburg, 26111 Oldenburg, Germany.

[f] Physikalisch-Technische Bundesanstalt Braunschweig und Berlin (PTB), Bundesallee 100, 38116 Braunschweig, Germany.

[g] Ikerbasque, Basque Foundation for Science, 48009 Bilbao, Spain.

† The authors contributed equally to this work.

* Correspondence to D. Medina: dana.medina@cup.lmu.de

Table of Contents

1. Characterization methods 3
Nuclear magnetic resonance (NMR) 3
X-ray diffraction 3
Scanning electron microscopy (SEM) 3
Transmission electron microscopy (TEM) 3
Nitrogen sorption 3
Thermogravimetric analysis (TGA) 4
Elemental analysis (EA) 4
Electrical conductivity measurements 4
Preparation of Fe-HBC-MOF pellets 4
Infrared spectroscopy (FTIR) 4
UV-Vis spectroscopy 5
Total hemispherical reflectance in geometry 8°/di 5
Zero-field ^{57}Fe Mössbauer spectroscopy 5
Electron paramagnetic resonance (EPR) spectroscopy 6
X-ray photoelectron spectroscopy 6
2. Experimental 7
General 7
Synthethesis procedures 7
Fe-HBC-MOF synthesis 9
General handling and stability tests 9
3. Characterization 10
4. Selected reports on electrically conducting 2D/3D MOFs. 18
References 20

1. Characterization methods

Nuclear magnetic resonance (NMR)

NMR spectra were recorded on Bruker AV400 and AV400 TR spectrometers. The chemical shifts are expressed in parts per million and calibrated using residual (undeuterated) solvent peaks as an internal reference (^{1}H-NMR: DMSO-d_6: 2.50; ^{13}C-NMR: acetone-d_6: 29.84). The data for ^{1}H-NMR spectra are reported as follows: s = singlet, d = doublet, t = triplet, q = quartet, m = multiplet.

X-ray diffraction

X-ray diffraction (XRD) analyses were performed on a Bruker D8 diffractometer in Bragg-Brentano geometry with Ni-filtered Cu K_α (λ = 1.54060 Å) radiation operating at 40 kV and 30 mA with a position-sensitive detector (LynxEye). PXRD measurements of Fe-HBC-MOF batches were conducted in transmission mode on a STOE Stadi MP diffractometer with a Cu Kα1 radiation source (λ = 1.54060 Å) operating at 40 kV and 40 mA. The diffractometer was equipped with a DECTRIS MYTHEN 1 K solid-state strip detector.

Scanning electron microscopy (SEM)

SEM images were recorded on a FEI Helios NanoLab G3 UC electron microscope with an acceleration voltage of 2 kV. Prior to SEM analysis, the samples were coated with a thin carbon layer by carbon fibre flash evaporation in high vacuum.

Transmission electron microscopy (TEM)

TEM images were collected on a FEI Titan Themis 60-300 microscope at an acceleration voltage of 300 kV. Powder samples were prepared by crushing the particles with a razor blade and subsequently depositing the powder onto a copper grid supporting a thin electron transparent carbon film.

Nitrogen sorption

Adsorption and desorption measurements were performed on an Autosorb 1 (Quantachrome instruments, Florida, USA) with nitrogen of 99.9999% purity at 77.3 K. The samples were activated under high vacuum at 120 °C for at least 12 h. Evaluation of adsorption and desorption isotherms was carried out with the AsiQwin v.3.01 (Quantachrome instruments, Florida, USA) software.

For BET calculations, pressure ranges of the nitrogen isotherms were chosen with the help of the BET assistant in the AsiQwin software. In accordance with the ISO recommendations, multipoint BET tags equal to or below the maximum in V x (1-p/p_0) in the semilogarithmic plot were chosen.

Thermogravimetric analysis (TGA)

Thermogravimetric analysis of the samples was performed using a NETZSCH STA 449C with a heating rate of 10 K min^{-1} and a heating range up to 900 °C under a stream of synthetic air with a gas flow rate of 25 mL min^{-1}.

Elemental analysis (EA)

The elemental analysis of the samples was performed on a Vario MICRO cube instrument (Elementar Analysensysteme GmbH, Germany).

Electrical conductivity measurements

Two-probe measurements of crystalline pellets were carried out under inert conditions with a Metrohm Autolab PGStat302N with an in house-built dc-conductivity measurement cell by recording I–V curves between −3 to +3 V by an Autolab 302N. The distance between the electrodes is equivalent to the thickness of the pellet, which was measured to be 130 µm. Van der Pauw measurements were conducted at room temperature under ambient conditions using an ECOPIA Model HMS-5300 Hall measurement setup at room temperature. Gold contact electrodes were placed in a square geometry with distances of about 2.4 mm on a pressed pellet of the MOF samples. Pellet thicknesses were measured with a slide gauge to be 130 µm.

Preparation of Fe-HBC-MOF pellets

MOF pellets with 1 cm diameter (obtained from several synthesis batches) for electrical conductivity measurements were fabricated with 60 mg of the respective MOF bulk material hand-tightened with a standard Paul-Weber KBr Press. An exemplary PXRD pattern of Fe-HBC-MOF pellet measured is shown in Fig. S4.

Infrared spectroscopy (FTIR)

ATR-Fourier-transform infrared (FTIR) spectra of powders were recorded on a Bruker Vertex 70 – RAM II instrument under nitrogen stream.

UV-Vis spectroscopy

UV-Vis spectra were recorded using a Perkin Elmer UV Vis/NIR Lambda 1050 spectrophotometer equipped with a 150 mm InGaAs integrating sphere. Diffuse reflectance spectra were collected with a Praying Mantis (Harrick) accessory and were referenced to barium sulphate powder as white standard.

Total hemispherical reflectance in geometry 8°/di

The total hemispherical reflectance was determined with an Agilent Cary 5000 double-beam spectrophotometer equipped with an external diffuse reflectance accessory eDRA. This system collects the total reflectance of diffuse reflecting specimens with an integration sphere of diameter d = 150 mm. The influx angle of the collimated radiation with respect to sample normal is 8°; the measurements described were performed in the gloss component included scheme. As the sample diameter was only 20 mm, the standard sample port of the sphere was reduced by a self-made $BaSO_4$-coated port reducer to 15 mm diameter and the eDRA optics for small spot measurements was used, resulting in an oval spot of about 10 mm height and 4 mm width. As a low spectral reflection of the sample was expected, it was measured in a two-step process relative to a calibrated PTFE white standard. In a first step a dark grey reflection standard with about 8% reflectance was measured against the white standard. Then the sample reflectance was determined against the grey standard. In both steps, the reference port was equipped with a nominal 25% reflectance standard. The zero-line of measurement was determined with open port by letting the influx radiation diffuse into an approx. 80 cm deep black cabinet with virtual zero back-reflection, shielded against the shaded laboratory environment.

Zero-field ^{57}Fe Mössbauer spectroscopy

Zero-field ^{57}Fe-Mössbauer spectra were recorded on a WissEl Mössbauer spectrometer (MRG-500) at a temperature of 77 K in constant acceleration mode. ^{57}Co/Rh was used as γ-radiation source. WinNormos for Igor Pro software was used for the quantitative evaluation of the spectral parameters (least-squares fitting to Lorentzian peaks). The minimum experimental line widths were 0.21 mm s^{-1} (full width at half maximum, FWHM). The temperature of the sample was controlled by an MBBC-HE0106 Mössbauer He/N_2 cryostat within an accuracy of +/–0.3 K. Least-square fitting of the Lorentzian signals was carried out with the “Mfit”

software, developed by Dr. Eckhard Bill (MPI Chemical Energy Conversion, Mülheim/Ruhr). The isomer shifts were reported relative to α-iron reference at 300 K.[1]

Electron paramagnetic resonance (EPR) spectroscopy

EPR spectra were recorded on a JEOL continuous-wave spectrometer JES-FA200, equipped with an X-band Gunn diode oscillator bridge, a cylindric mode cavity, and a helium cryostat. The samples were measured as a solid under nitrogen in quartz glass EPR tubes at 95, and 293 K. The spectra shown were measured using the following parameters: microwave frequency = 8.959 GHz, modulation amplitude 1.00 mT, microwave power 1.0 mW, modulation frequency 100 kHz, time constant of 0.1 s. Data analysis and simulation of the data was performed using the software eview and esim, written by Dr. Eckhard Bill (MPI Chemical Energy Conversion, Mülheim/Ruhr)[2], based on a spin-Hamiltonian description of the electronic ground state:

$$\hat{H} = D\left(\hat{S}_z^2 - \frac{1}{3}S(S+1) + \frac{E}{D}\left(\hat{S}_x^2 - \hat{S}_y^2\right)\right) + \mu_B \underline{g}\vec{S} \;.$$

Here, S represents the total spin quantum number of the coupled system, D and E/D are the axial and rhombic zero-field parameters, respectively, and g is the g-matrix. Calculations are based on the S = 5/2 routines developed by Gaffney and Silverstone.[3] EPR line widths, W, are given in units of $\cdot\ 10^{-4}$ cm^{-1} / GHz at full-width-half-maximum (FWHM).

X-ray photoelectron spectroscopy

XPS was performed using an ESCALAB 250 Xi instrument (Thermo Fisher, East Grinstead, UK) with monochromatized Al Kα (hv = 1486.6 eV) radiation focused to a spot of 500 µm diameter at the surface of samples. Spectra were measured with pass energies of 200 eV for survey scans and 10 eV for high-resolution regions. Charging was compensated by the use of an internal electron flood gun. Peak fitting was performed by the software Avantage, version 5.9904 (Thermo Fisher) using a Shirley background ("Smart Shirley") and a convolution of Gaussian and Lorentzian functions for each signal component.[4] All spectra were referenced to remaining adventitious carbon at 284.8 eV.

2. Experimental

General

All materials were purchased from Sigma Aldrich, Acros or TCI Europe in the common purities purum, puriss or reagent grade. The materials were used as received without additional purification and handled in air unless otherwise noted.

The water utilized in the synthesis was subjected to a Merck-Milipore Mili-Q purification system prior to use.

Synthesis procedures

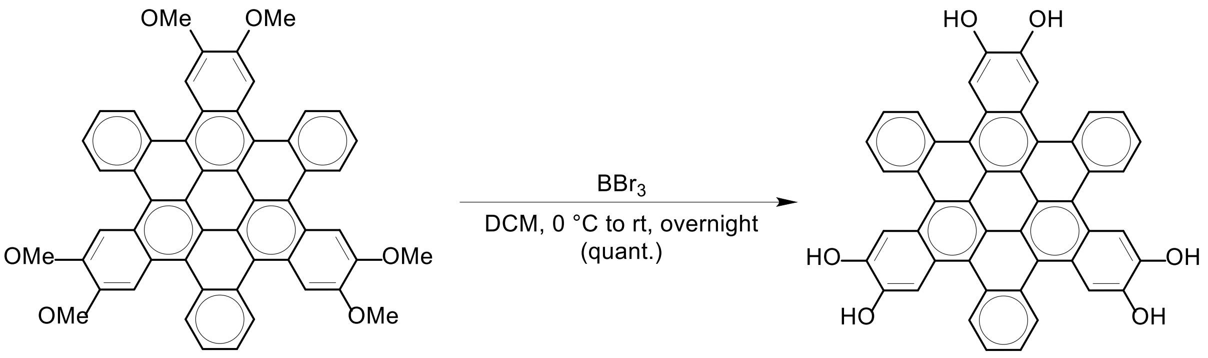

2,3,10,11,18,19-hexahydroxy-*cata*-hexabenzocoronene (*c*-HBC) was synthesized according to a reported procedure.[5] 2,3,10,11,18,19-hexamethoxy-*cata*-hexabenzocoronene (30 mg, 0.038 mmol) was dissolved in 3 mL of dry dichloromethane. The solution was cooled down to 0 °C and 3 mL of BBr_3 (1M solution in dichloromethane) were added. Afterwards, the solution was allowed to warm up to room temperature and stirred under argon overnight. After the reaction was completed, water was added to quench the reaction and the solvent was partly removed using an argon flow. The resulting precipitate was filtered and finally dried under reduced pressure to give *c*-HBC as a light green solid in a quantitative yield.

^{1}H NMR (400 MHz, $(CD_3)_2SO$): δ= 9.98 (s, 6H), 9.20-9.18 (m, 6H) 8.58 (s, 6H), 7.88-7.86 (m, 6H) ppm; ^{13}C NMR (100 MHz, $(CD_3)_2CO$): δ= 146.84, 130.81, 129.31, 127.07, 125.87, 125.33, 121.09, 113.73 ppm.

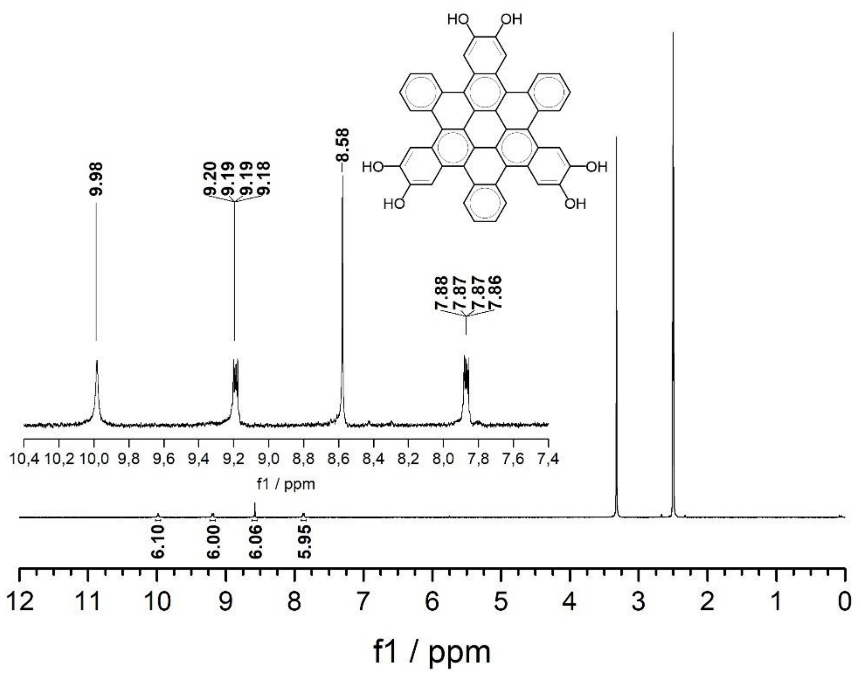

Fig. S1 ^{1}H NMR spectrum of *c*-HBC in DMSO-d_6 at room temperature.

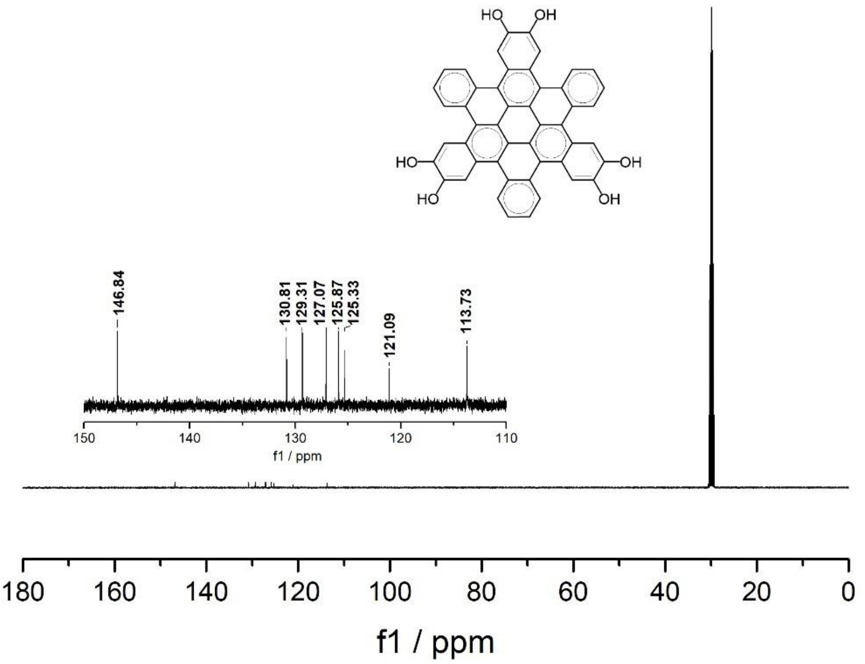

Fig. S2 ^{13}C NMR spectrum of *c*-HBC in acetone-d_6 at room temperature.

Fe-HBC-MOF synthesis

Freshly synthesized *c*-HBC (11.0 mg, 15.8 mmol), $Fe(BF_4)_2{\cdot}6H_2O$ (21.0 mg, 62.2 mmol) and tetrabutylammoniumnitrate (10.0 mg, 41.2 mmol) were suspended in DMF (1.75 mL), NMP (125 µL), MeOH (125 µL), H_2O (125 µL) and mesitylene (50 µL) solvent mixture in a culture tube under argon atmosphere. The resulting mixture was sonicated until complete dissolution of the reactants was achieved. The reactor with the reaction mixture was kept at 120 °C for 3 days. The resulting precipitate was washed with DMF (3 x 20 mL), water (1 x 20 mL), methanol (3 x 20 mL) and activated at 120 °C to remove the solvent residues to afford a black crystalline powder. CHN elemental analysis: Anal. Found (%): C, 51.7; H, 3.76; N, 1.86. Calc. for $((CH_3)_2NH_2)_2\,[Fe_3O(HBC^{6-})\,(HBC^{\cdot 3-})]{\cdot}37$ Water·1 DMF (%): C, 51.6; H, 5.59; N, 1.75.

General handling and stability tests

The crystallinity of the Fe-HBC-MOF was found to be dependent on the quality of the *c*-HBC linker and on the exposure time to air. We note that highly crystalline batches remain stable for at least one month under ambient conditions in a closed vessel.

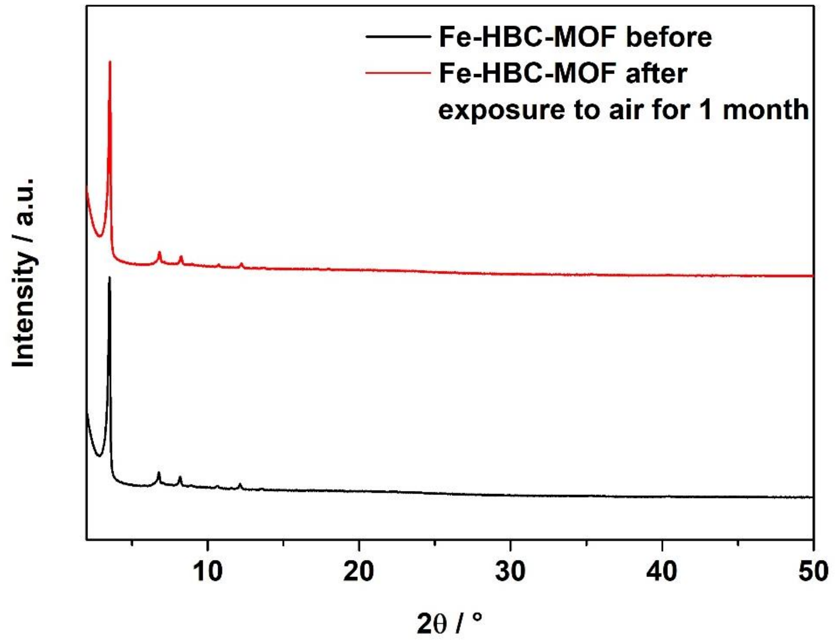

Fig. S3 PXRD pattern of a Fe-HBC-MOF sample, freshly prepared (black) and aged under ambient air in a closed container (red).

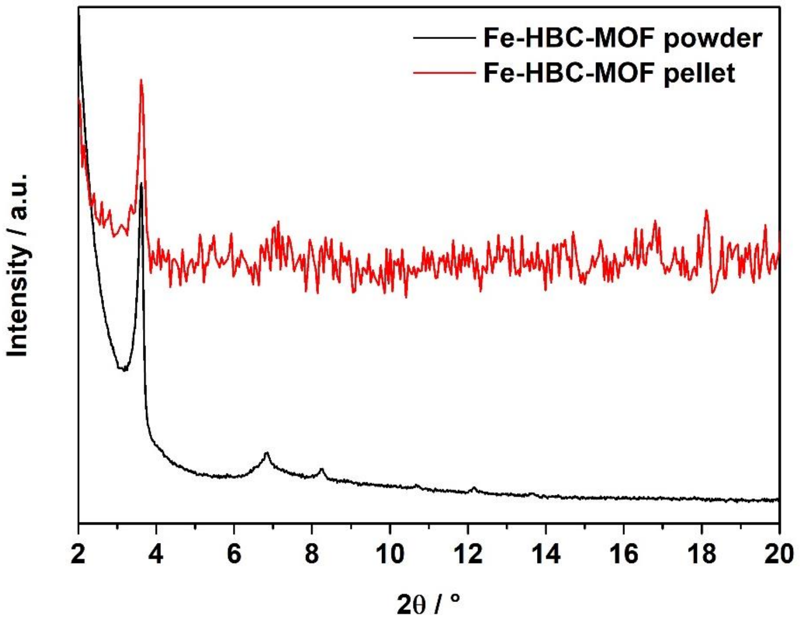

Fig. S4 PXRD pattern of the Fe-HBC-MOF (same batch) before (black) and after pressing a pellet (red).

3. Characterization

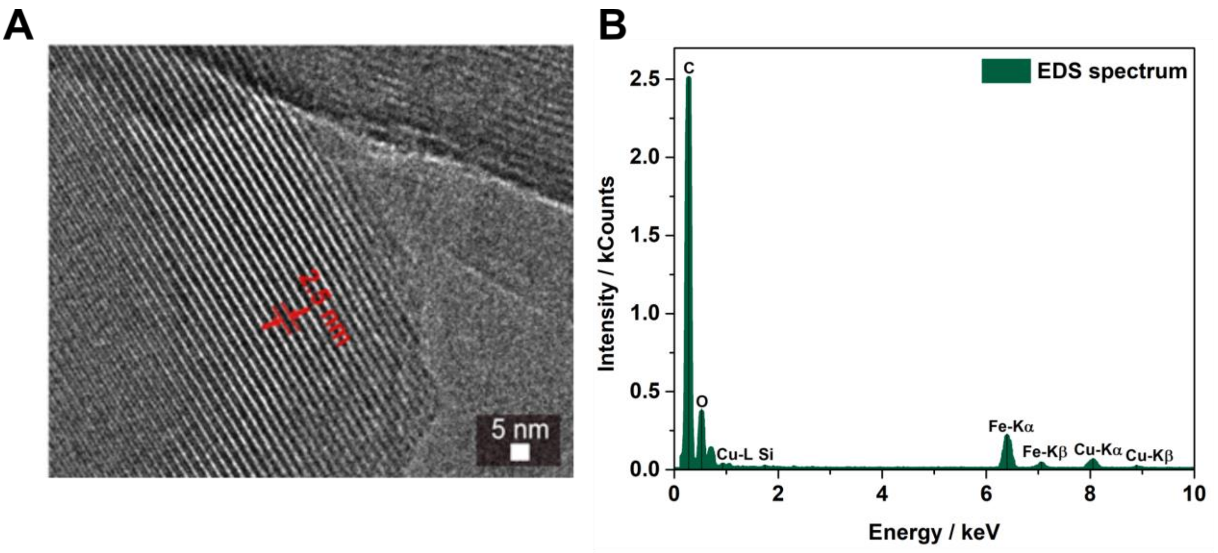

Fig. S5 TEM image of the Fe-HBC-MOF, showing an interplanar distance of 2.5 nm, which is in good accordance with the 111 reflection in the PXRD (A), EDS spectrum of Fe-HBC-MOF showing the presence of Fe, O and C (B).

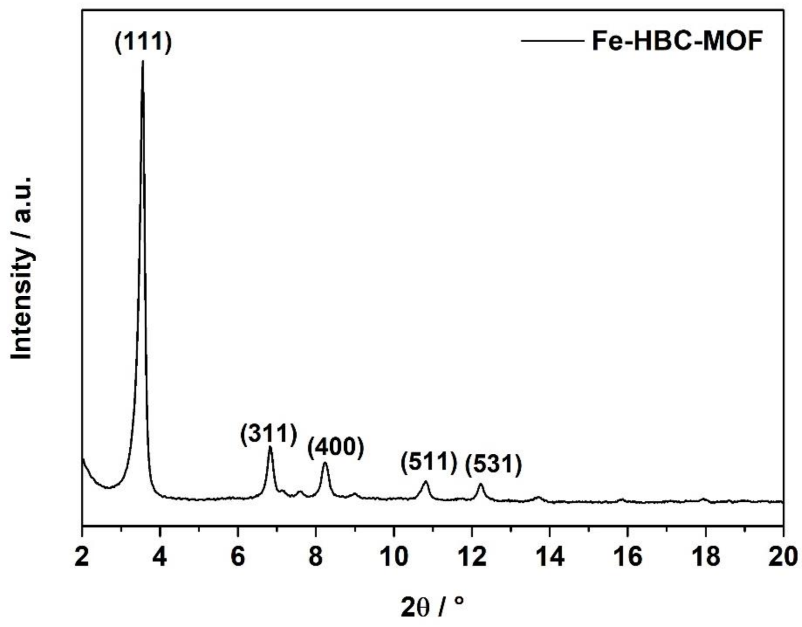

Fig. S6 Indexed PXRD pattern of the Fe-HBC-MOF measured as powder at room temperature.

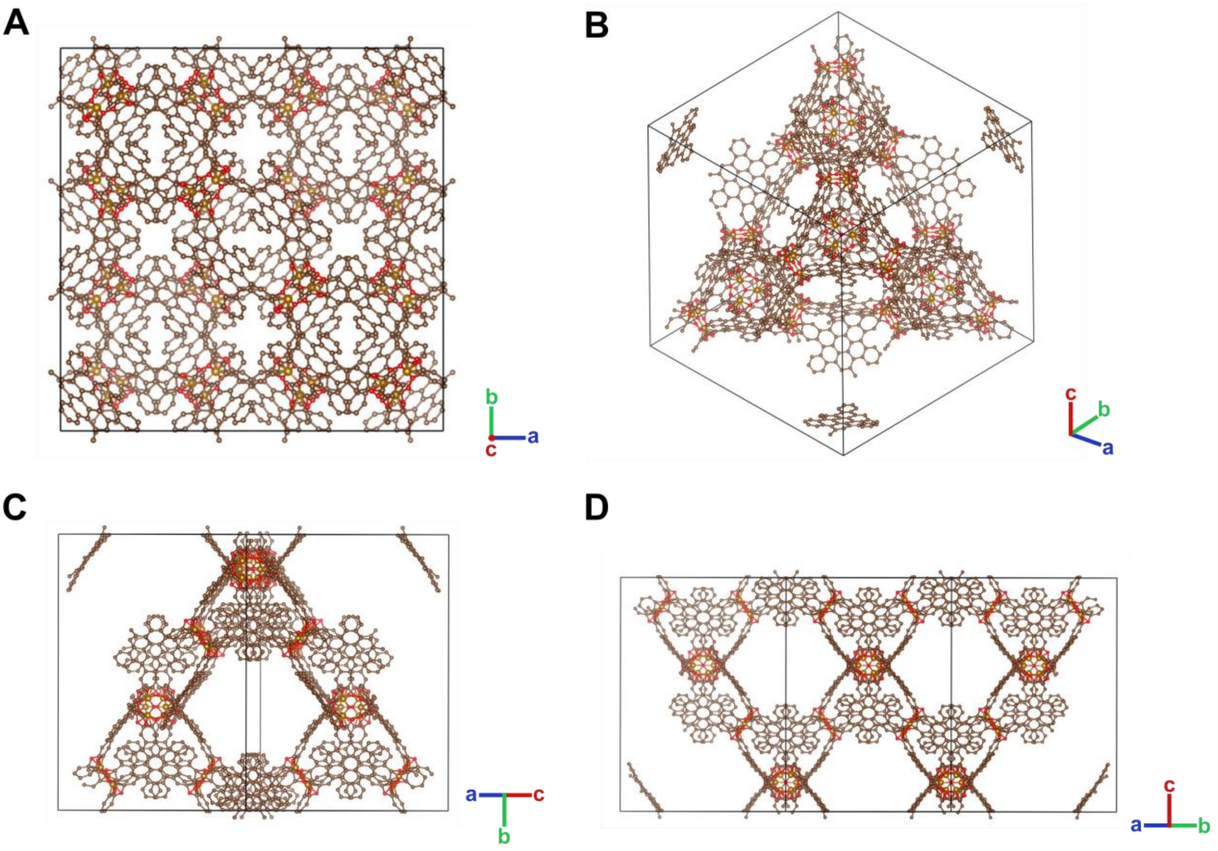

Fig. S7 Images of the simulated model structure of the Fe-HBC-MOF viewed along the c-axis (A), display of the open supertetrahedron (B), view on the pore system (C) and the arrangement of c-HBC ligands in connected supertetrahedra units (D).

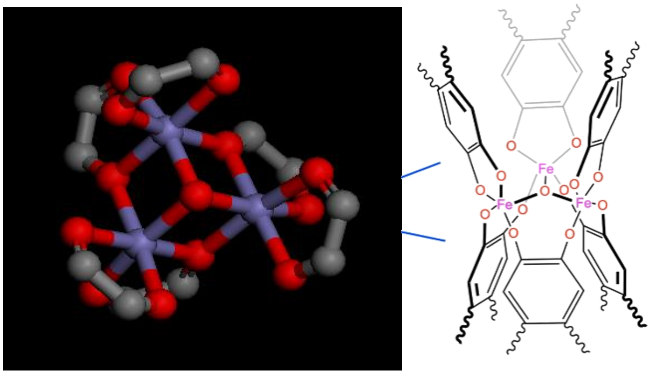

Fig. S8 Visualization of the iron oxo cluster node. In a trimeric geometry, three iron bis-catecholate complexes are stabilized by a central oxygen atom.

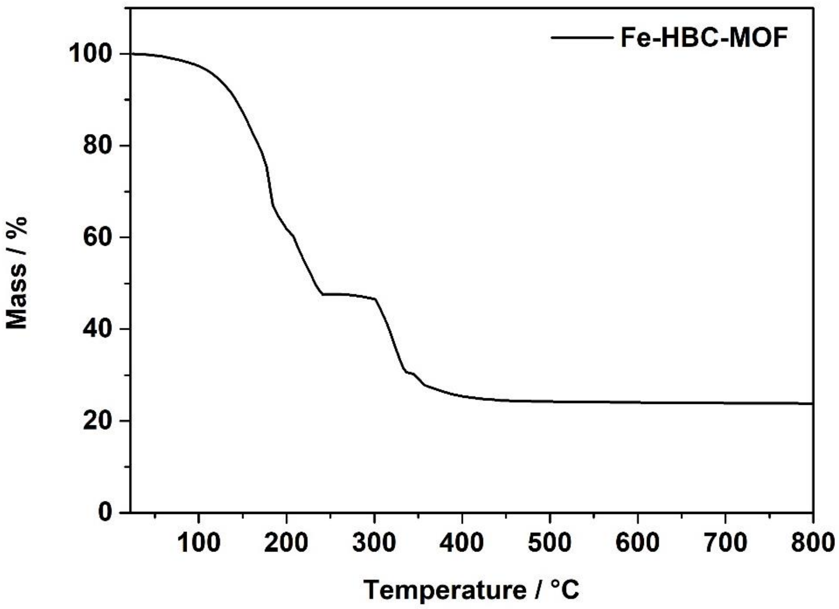

Fig. S9 Thermogravimetric analysis (TGA) of Fe-HBC-MOF under a stream of synthetic air. First mass loss observed at 110 °C is attributed to the loss of solvent molecules and structural degradation of the framework followed by a second mass loss observed at 300 °C attributed to the final framework decomposition, to give a metal oxide mass residue.

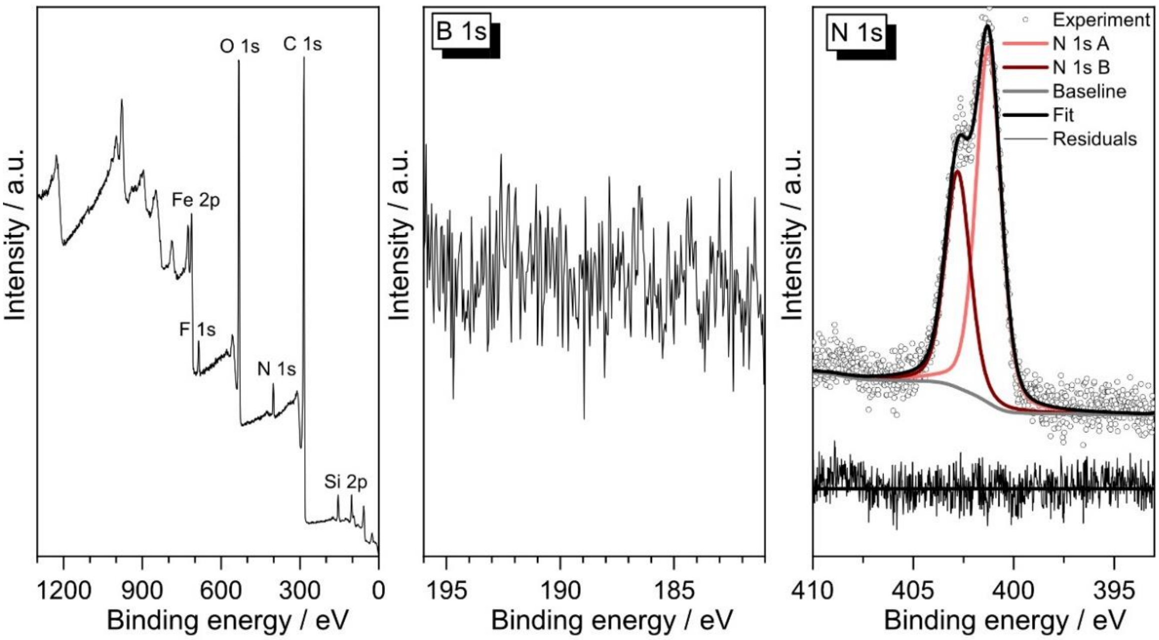

Fig. S10 XPS analysis of Fe-HBC-MOF for binding energy of the survey scan, B 1s and N 1s. Survey scan of the sample (left). No boron impurities coming from the iron precursor salt $Fe(BF_4)_2 \cdot 6H_2O$ could be detected (centre). Two types of nitrogen, which are not nitrate, were detected (right). We attribute the signals to residual DMF and NMP in the sample.

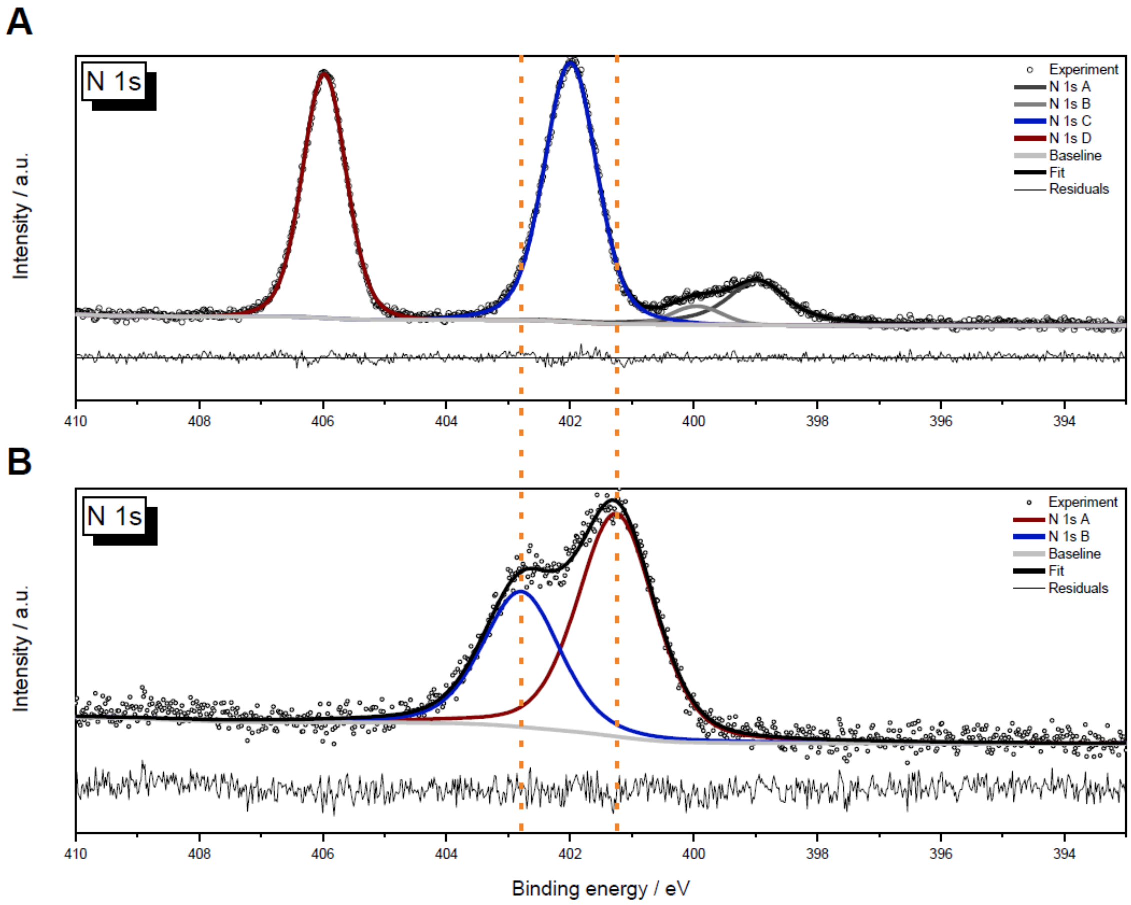

Fig. S11 High-resolution XP spectra of the N 1s region of tetrabutylammonium nitrate (A) and Fe-HBC-MOF (B). The N 1s components in Fe-HBC-MOF correspond to nitrate (406.0 eV) and the quaternary N-atom of the tetrabutylammonium ion (402.0 eV). The signal components at 399.0 eV and 400.0 eV are unknown contaminations of tetrabutylammonium nitrate that do not occur in the MOF (B). The two components in the N 1s spectrum of Fe-HBC-MOF are attributed to residual DMF and NMP used in the synthesis.

The C 1s spectra contain the prominent peak for the unsubstituted aromatic carbon atoms in the center of the catecholate ligand (component A). The shake-up feature for aromatic compounds (component E) is clearly visible. Spectral features of the substituted carbon atoms of the ligand occur at higher binding energies. The exact assignment is challenging due to the occurrence of solvent residues and ubiquitous carbon-containing surface contamination for samples handled under atmosphere, which contribute to similar binding energies as expected for the organic ligand. Due to the surface-sensitive character of XPS, this thin contamination layer contributes over-proportionally to the observed spectrum.

There are two signals in the O 1s spectrum. The component A at E_B = 531.8 eV is assigned to Fe-O-C. Compared to iron oxides, this component is shifted to higher binding energies as observed in other Fe-MOFs.[6] The binding energy of component B is assigned to uncoordinated C-OH groups as also found in polymers such as polydopamine.[7] However, this

is also the binding energy at which carbonyl groups of solvent residues similar to polymers with carbonyl groups,[8] C-O groups from ubiquitous surface contaminations and adsorbed water [9] would contribute to the spectrum.

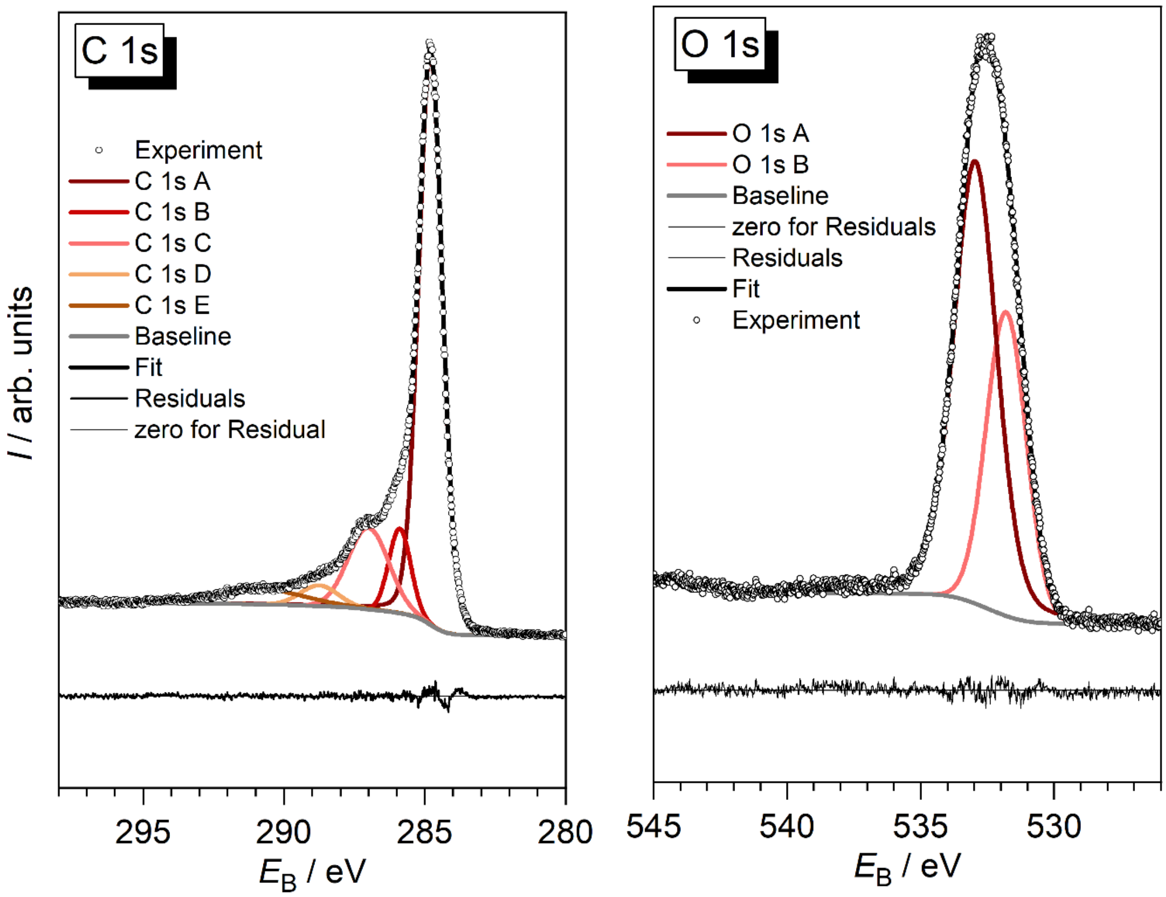

Fig. S12 High-resolution XP spectra of the C 1s and O 1s regions of the Fe-HBC-MOF.

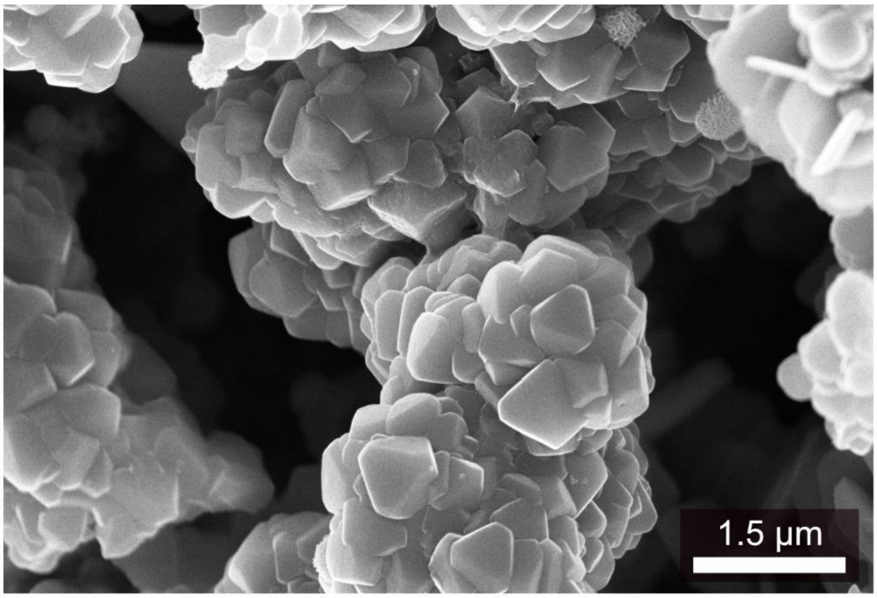

Fig. S13 Low magnification SEM image of truncated-octahedron crystallites of Fe-HBC-MOF showing the overall homogeneity of the MOF powder.

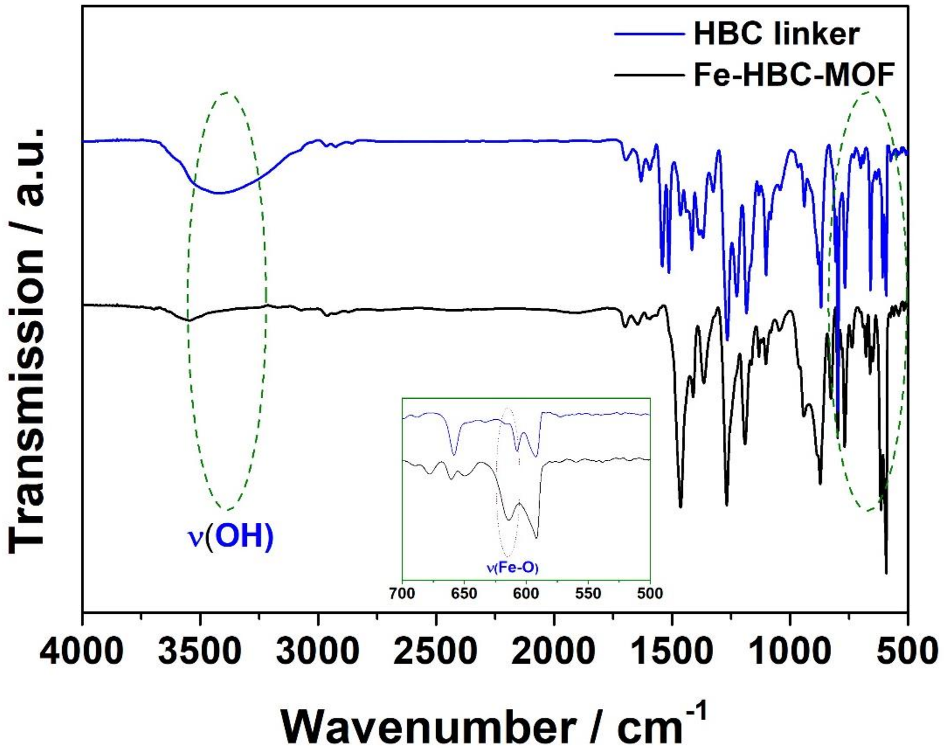

Fig. S14 Fourier-transform infrared spectrum (FTIR) of the *c*-HBC ligand (blue) and a crystalline and activated Fe-HBC-MOF (black) powder sample. The vibration mode attributed to the hydroxyl groups of the *c*-HBC linker ν(OH) at 3400 cm^{-1} is evidently attenuated in the respective spectrum of the synthesized MOF. The green inset displays an enlargement of the spectral range between 700 and 500 cm^{-1}, showing a ν(Fe–O) band at 615 cm^{-1} attributed to the formation of an iron-oxygen bond.

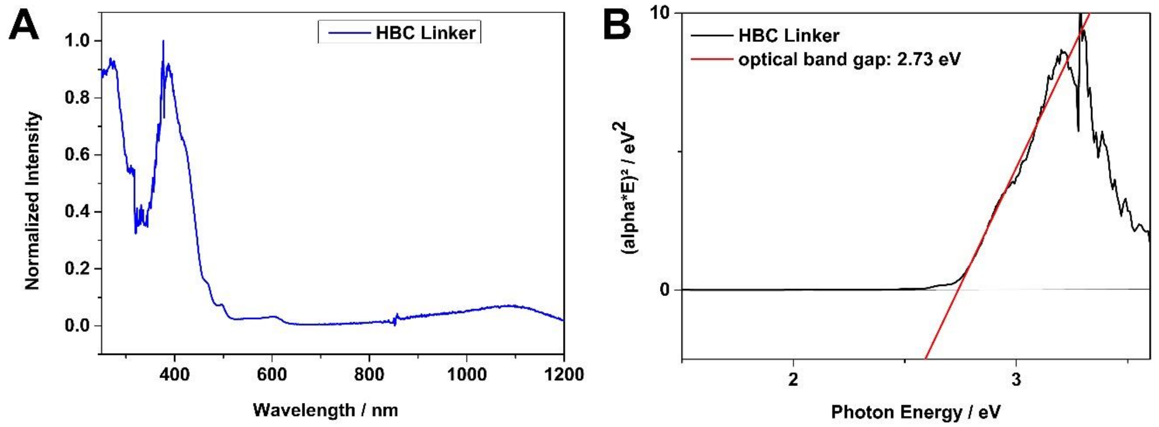

Fig. S15 UV-Vis-NIR spectrum of the *c*-HBC ligand (A). Tauc plot of the *c*-HBC ligand (B), indicating a direct band gap of 2.7 eV.

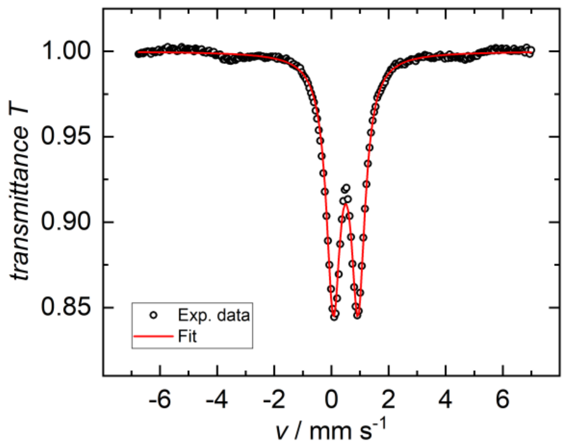

Fig. S16 Zero-field ^{57}Fe Mössbauer spectrum of Fe-HBC-MOF recorded at 77 K. Black circles: experimental data, red line: best fit to experimental data with parameters: δ = 0.50 mm s^{-1}, ΔE_Q = 0.87 mm s^{-1}, Γ_{FWHM} = 0.59 mm s^{-1}.

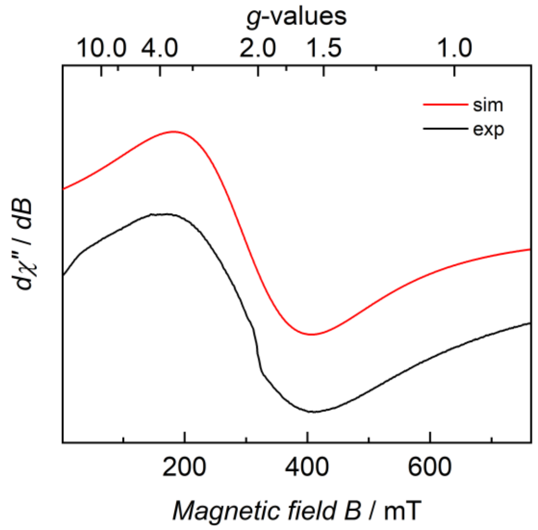

Fig. S17 CW X-band EPR spectrum of Fe-HBC-MOF recorded as a solid at 293 K (black trace), and its simulation (red trace). Experimental conditions: microwave frequency ν = 8.947 GHz, modulation width = 1.0 mT, microwave power = 1.0 mW, modulation frequency = 100 kHz, time constant = 0.1 s. Simulation parameters: effective *g*–value g_{iso} = 2.17, linewidth W_{iso} = 255 · 10^{-4} cm^{-1} / GHz, pseudo-Voigt lines used with ratios (Lorentz = 0, Gauss = 1) V_{iso} = 0.00.

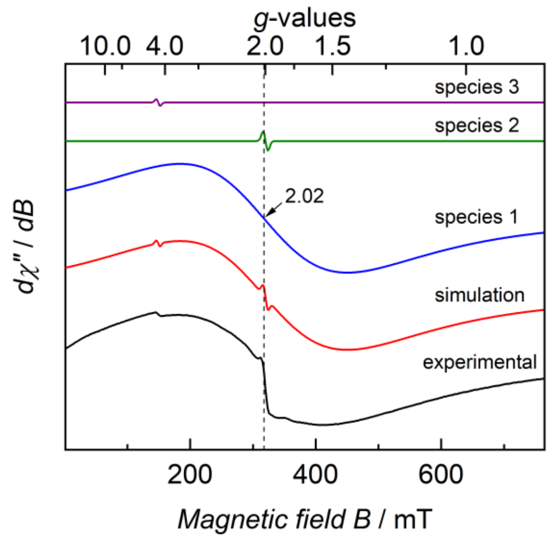

Fig. S18 CW X-band EPR spectrum of Fe-HBC-MOF recorded as a solid at 95 K (black trace), its simulation (red trace), main species (blue trace) plus very minor impurities (green and purple trace) in the sub‰ range. Experimental conditions: microwave frequency ν = 8.968 GHz, modulation width = 1.0 mT, microwave power = 1.0 mW, modulation frequency = 100 kHz, time constant = 0.1 s. Simulation parameters for species 1: effective *g*–value g_{iso} = 2.02, linewidths W_{iso} = 280 · 10^{-4} cm^{-1} / GHz, pseudo-Voigt lines used with ratios (Lorentz = 0, Gauss = 1) V_{iso} = 0.00. Simulation parameters for species 2: effective *g*–value g_{iso} = 2.00, linewidth W_{iso} = 8.00 · 10^{-4} cm^{-1} / GHz, pseudo-Voigt lines used with ratios (Lorentz = 0, Gauss = 1) V_{iso} = 1.00. Simulation parameters for species 3: effective *g*–value g_{iso} = 4.30, linewidth W_{iso} = 14.0 · 10^{-4} cm^{-1}/ GHz, pseudo-Voigt lines used with ratios (Lorentz = 0, Gauss = 1) V_{iso} = 1.00.

4. Selected reports on electrically conducting 2D/3D MOFs.

Tab. S1 Intrinsic electrical conductivity of iron- and coronene-based MOFs.

	Material	Dimensionality	Porosity	Method	El.conductivity [S cm^{-1}]
Sun *et al.*[10]	Fe-DOBDC	2D	241 m^2 g^{-1}	Pellet, 2-probe	$3.2{\cdot}10^{-7}$
Aust *et al.*[11]	Fe-BDDTC	Structure is not reported	237 m^2 g^{-1}	Pellet, Van der Pauw	$5{\cdot}10^{-3}$
Wu *et al.*[12]	Fe-THBQ	3D	163 m^2 g^{-1}	Pellet, 4-probe	$2.7{\cdot}10^{-4}$
Mähringer *et al.*[13]	Fe-HHTP	3D	1400 m^2 g^{-1}	Pellet, Van der Pauw	$5.6{\cdot}10^{-3}$
Gupta *et al.*[14]	Fe-DHBQ	3D	556 m^2 g^{-1}	Pellet, 2-probe	$1.2{\cdot}10^{-2}$

Darago *et al.*[15]	Fe-DHBQ	3D	Non-porous	Pellet, 2-probe	0.16
Dong *et al.*[16]	Fe-PTC	2D	210 m^2 g^{-1}	Pellet, Van der Pauw	0.1
Chen *et al.*[17]	Co-PTC	2D	Not reported	Pellet, 4-probe	45
Chen *et al.*[18]	Ni-PTC	2D	Not reported	Pellet, 4-probe	9
Xing *et al.*[19]	Cu-HBC-12O	2D	228 m^2 g^{-1}	Pellet, 2-probe	$3.3 \cdot 10^{-2}$
Zhang *et al.*[20]	Cu-6FcHBC	2D	426.9 m^2 g^{-1}	Single-crystal, 2-probe	5.2
DOBDC= 2,5-dihydroxybenzene-1,4-dicarboxylate, BDDTC= 1,4-di(dithiocarboxylate), THBQ= tetrahydroxy-1,4-benzoquinone, HHTP= 2,3,6,7,10,11-hexahydroxytriphenylene, DHBQ= 2,5-dihydroxy-1,4-benzoquinone, PTC= 1,2,3,4,5,6,7,8,9,10,11,12-perthiolated coronene, HBC-12OH=2,3,6,7,10,11,14,15,18,19,22,23-dodecahydroxy-cata-hexabenzocoronene, 6FcHBC=2,3,10,11,18,19-hexafluoro-6,7,14,15,22,23-hexahydroxy-cata-hexabenzocoronene.					

Tab. S2 Selected conductive 2D/3D MOFs in diverse fields of application.

**Material**	**Dimensionality**	**El. conductivity**	**deposition substrate**	**Application**
Cu-CAT-1	2D	2.1 S cm^{-1} (single-crystal, four-probe)[21]	carbon fibre paper	solid-state supercapacitor[22]
			Si/SiO_2 , sapphire or glass substrate	chemiresistive sensor[23]
Ni-CAT-1	2D	10^{-3} S cm^{-1} (oriented film, van der Pauw)[24]	ITO substrate	photovoltaic device[24]
$Cu_3(HITP)_2$	2D	0.2 S cm^{-1} (pellet, two-probe)[25]	gold electrodes	chemiresistive sensor[26]
			shrinkable polymer films, cotton fibres	protective systems on flexible fibres[27]
$Co_3(HITP)_2$	2D	11.5 S cm^{-1} (pellet, van der Pauw)[28]	carbon cloth (CC)	electrocatalysis[28]
$Ni_3(HITP)_2$	2D	2.0 S cm^{-1} (pellet, two-probe)[29]	cellulose nanofibers	flexible and foldable supercapacitor[30]

Cu-BHT	2D	1580 S cm^{-1} (film, four-probe) [31]	multiwall CNTs	lithium-ion batteries [32]
Cu-TAPT	3D	4 S cm^{-1} (single-crystal, 2-probe)[33]	super P carbon black	sodium-ion batteries[33]
CAT-1= HHTP= 2,3,6,7,10,11-hexahydroxytriphenylene, HITP=2,3,6,7,10,11-hexaiminotriphenylene, BHT= 1,2,3,4,5,6-benzenehexathiol, TAPT= 2,3,7,8-tetraaminophenazine-1,4,6,9-tetraone.				

References

[1] a) E. Bill, *Mössbauer Program Mfit*, **2019**; b) E. Bill, *Mössbauer Program Mcal*, **2019**.

[2] a) E. Bill, *EPR Program eview*, **2019**; b) E. Bill, *EPR Program esim*, **2019**.

[3] B. J. Gaffney, H. J. Silverstone in *EMR of Paramagnetic Molecules* (Eds.: L. J. Berliner, J. Reuben), Springer US, Boston, MA, **1993**, pp. 1–57.

[4] a) R. P. Gupta, S. K. Sen, *Phys. Rev. B* **1974**, *10*, 71; b) R. P. Gupta, S. K. Sen, *Phys. Rev. B* **1975**, *12*, 15; c) A. P. Grosvenor, B. A. Kobe, M. C. Biesinger, N. S. McIntyre, *Surf. Interface Anal.* **2004**, *36*, 1564.

[5] M. Martínez-Abadía, C. T. Stoppiello, K. Strutynski, B. Lerma-Berlanga, C. Martí-Gastaldo, A. Saeki, M. Melle-Franco, A. N. Khlobystov, A. Mateo-Alonso, *J. Am. Chem. Soc.* **2019**, *141*, 14403.

[6] a) Z. Wang, Y. Fang, Y. Yang, B. Qiu, T. Zhang, H. Li, W. Zhao, *J. Environ. Chem. Eng.* **2022**, *10*, 107876; b) L. Zhang, W. Liu, W. Shi, X. Xu, J. Mao, P. Li, C. Ye, R. Yin, S. Ye, X. Liu et al., *Chem. Eur. J.* **2018**, *24*, 13792; c) S. Zhang, Y. Zhuo, C. I. Ezugwu, C. Wang, C. Li, S. Liu, *Environ. Sci. Technol.* **2021**, *55*, 8341.

[7] a) R. Luo, L. Tang, J. Wang, Y. Zhao, Q. Tu, Y. Weng, R. Shen, N. Huang, *Colloids Surf. B.* **2013**, *106*, 66; b) X. Han, X. Chen, M. Yan, H. Liu, *Particuology* **2019**, *44*, 63.

[8] B. V. Christ, *Handbook of Monochromatic XPS Spectra*, Vol. 4, XPS International, Salem, Oregon, **2019,** p.22.

[9] M. Marioli, P. Luo., T. Kuwana, *Anal. Chim. Acta* **1991**, *248*, 117.

[10] L. Sun, C. H. Hendon, M. A. Minier, A. Walsh, M. Dincă, *J. Am. Chem. Soc.* **2015**, *137*, 6164.

[11] M. Aust, M. I. Schönherr, D. P. Halter, L. Schröck, T. Pickl, S. N. Deger, M. Z. Hussain, A. Jentys, R. Bühler, Z. Zhang et al., *Inorg. Chem.* **2024**, *63*, 129.

[12] X. Wu, Y. Qiu, Z. Chen, B. Guan, X. Hao, A. I. Rykov, Y. Sun, L. Liu, Y. Zou, J. Sun et al., *Angew. Chem. Int. Ed.* **2020**, *59*, 20873.

[13] A. Mähringer, M. Döblinger, M. Hennemann, C. Gruber, D. Fehn, P. I. Scheurle, P. Hosseini, I. Santourian, A. Schirmacher, J. M. Rotter et al., *Angew. Chem. Int. Ed.* **2021**, *60*, 18065.

[14] S. Gupta, H. Tanaka, K. Fuku, K. Uchida, H. Iguchi, R. Sakamoto, H. Kobayashi, Y. Gambe, I. Honma, Y. Hirai et al., *Inorg. Chem.* **2023**, *62*, 6306.

[15] L. E. Darago, M. L. Aubrey, C. J. Yu, M. I. Gonzalez, J. R. Long, *J. Am. Chem. Soc.* **2015**, *137*, 15703.

[16] R. Dong, Z. Zhang, D. C. Tranca, S. Zhou, M. Wang, P. Adler, Z. Liao, F. Liu, Y. Sun, W. Shi et al., *Nat. Commun.* **2018**, *9*, 2637.

[17] Z. Chen, Y. Cui, C. Ye, L. Liu, X. Wu, Y. Sun, W. Xu, D. Zhu, *Chem. Eur. J.* **2020**, *26*, 12868.

[18] Z. Chen, Y. Cui, Y. Jin, L. Liu, J. Yan, Y. Sun, Y. Zou, Y. Sun, W. Xu, D. Zhu, *J. Mater. Chem. C* **2020**, *8*, 8199.

[19] G. Xing, J. Liu, Y. Zhou, S. Fu, J.-J. Zheng, X. Su, X. Gao, O. Terasaki, M. Bonn, H. I. Wang et al., *J. Am. Chem. Soc.* **2023**, *145*, 8979.

[20] J. Zhang, G. Zhou, H.-I. Un, F. Zheng, K. Jastrzembski, M. Wang, Q. Guo, D. Mücke, H. Qi, Y. Lu et al., *J. Am. Chem. Soc.* **2023**, *145*, 23630.

[21] M. Hmadeh, Z. Lu, Z. Liu, F. Gándara, H. Furukawa, S. Wan, V. Augustyn, R. Chang, L. Liao, F. Zhou et al., *Chem. Mater.* **2012**, *24*, 3511.

[22] W.-H. Li, K. Ding, H.-R. Tian, M.-S. Yao, B. Nath, W.-H. Deng, Y. Wang, G. Xu, *Adv Funct Materials* **2017**, *27*.

[23] M.-S. Yao, X.-J. Lv, Z.-H. Fu, W.-H. Li, W.-H. Deng, G.-D. Wu, G. Xu, *Angew. Chem. Int. Ed.* **2017**, *56*, 16510.

[24] A. Mähringer, A. C. Jakowetz, J. M. Rotter, B. J. Bohn, J. K. Stolarczyk, J. Feldman, T. Bein, D. D. Medina, *ACS Nano* **2019**, *13*, 6711.

[25] M. G. Campbell, D. Sheberla, S. F. Liu, T. M. Swager, M. Dincă, *Angew. Chem. Int. Ed.* **2015**, *54*, 4349.

[26] M. G. Campbell, S. F. Liu, T. M. Swager, M. Dincă, *J. Am. Chem. Soc.* **2015**, *137*, 13780.

[27] a) M. K. Smith, K. E. Jensen, P. A. Pivak, K. A. Mirica, *Chem. Mater.* **2016**, *28*, 5264; b) A. M. Eagleton, M. Ko, R. M. Stolz, N. Vereshchuk, Z. Meng, L. Mendecki, A. M. Levenson, C. Huang, K. C. MacVeagh, A. Mahdavi-Shakib et al., *J. Am. Chem. Soc.* **2022**, *144*, 23297.

[28] D. Xing, Y. Wang, P. Zhou, Y. Liu, Z. Wang, P. Wang, Z. Zheng, H. Cheng, Y. Dai, B. Huang, *Appl. Catal. B: Environ.* **2020**, *278*, 119295.

[29] D. Sheberla, L. Sun, M. A. Blood-Forsythe, S. Er, C. R. Wade, C. K. Brozek, A. Aspuru-Guzik, M. Dincă, *J. Am. Chem. Soc.* **2014**, *136*, 8859.

[30] S. Zhou, X. Kong, B. Zheng, F. Huo, M. Strømme, C. Xu, *ACS Nano* **2019**, *13*, 9578.

[31] X. Huang, P. Sheng, Z. Tu, F. Zhang, J. Wang, H. Geng, Y. Zou, C. Di, Y. Yi, Y. Sun et al., *Nat. Commun.* **2015**, *6*, 7408.

[32] Z. Wu, D. Adekoya, X. Huang, M. J. Kiefel, J. Xie, W. Xu, Q. Zhang, D. Zhu, S. Zhang, *ACS Nano* **2020**, *14*, 12016.

[33] K. Fan, J. Li, Y. Xu, C. Fu, Y. Chen, C. Zhang, G. Zhang, J. Ma, T. Zhai, C. Wang, *J. Am. Chem. Soc.* **2023**, *145*, 12682.
